# A multi-center analysis of visual outcomes following open globe injury

**DOI:** 10.1038/s41598-024-67564-y

**Published:** 2024-07-18

**Authors:** Jason A. Greenfield, Davina A. Malek, Shruti Anant, Michael Antonietti, Alessandro Jammal, Alicia Casella, Sarah C. Miller, Kristine Wang, Bita Momenaei, Karen Lee, Hana A. Mansour, Grant A. Justin, Kevin G. Makhoul, Racquel A. Bitar, Alice C. Lorch, Grayson W. Armstrong, Taku Wakabayashi, Yoshihiro Yonekawa, Fasika Woreta, Kara Cavuoto

**Affiliations:** 1 Bascom Palmer Eye Institute, University of Miami Miller School of Medicine, 900 NW 17Th Street, Miami, FL 33136 USA; 2 Wilmer Eye Institute, Johns Hopkins Hospital, Baltimore, MD USA; 3 Wills Eye Hospital, Jefferson Health, Philadelphia, PA USA; 4 Duke Department of Ophthalmology, Duke University, Durham, NC USA; 5 Massachusetts Eye and Ear, Harvard Medical School, Boston, MA USA; 6 KU Eye Center, University of Kansas Medical Center, Prairie Village, KS USA

**Keywords:** Open globe injury, Ocular trauma, Etiology, Visual outcome, Prognosis, Risk factors, Outcomes research, Eye manifestations

## Abstract

The purpose of this study was to examine how demographics, etiology, and clinical examination findings are related to visual outcomes in subjects with open globe injury (OGI) across a large and generalizable sample. A retrospective cohort analysis was performed using data collected from the electronic medical records of four tertiary university centers for subjects with OGI presenting from 2018 to 2021. Demographic information, injury mechanisms, clinical exam findings, visual acuity (VA) at presentation and most recent follow-up were recorded. In subjects with bilateral OGIs, only right eyes were included. A modified ocular trauma score (OTS) using presenting VA, the presence of perforating injury, OGI, and afferent pupillary defect was calculated. The risk of subjects’ demographic characteristics, ocular trauma etiology, clinical findings and modified OTS on the presence of monocular blindness at follow-up were assessed using univariable and multivariable regression models. 1426 eyes were identified. The mean age was 48.3 years (SD: ± 22.4 years) and the majority of subjects were men (N = 1069, 75.0%). Univariable analysis demonstrated that subjects of Black race were 66% (OR: 1.66 [1.25–2.20]; *P* < 0.001) more likely to have monocular blindness relative to White race at follow-up. OTS Class 1 was the strongest predictor of blindness (OR: 38.35 [21.33–68.93]; *P* < 0.001). Based on multivariable analysis, lower OTS category (OTS Class 1 OR: 23.88 [16.44–45.85]; *P* < 0.001) moderately predicted visual outcomes (R^2^ = 0.275, *P* < 0.001). OGI has many risks of poor visual outcome across patient groups that vary by demographic category, mechanism of injury, and clinical presentation. Our findings validate that a modified OTS remains a strong predictor of visual prognosis following OGI in a large and generalizable sample.

Approximately 1.6 million of the 55 million ocular traumas that occur annually lead to blindness, making ocular trauma the leading cause of monocular blindness globally^1^. Of these, 200,000 are open globe injuries (OGI) due to either a laceration or rupture^2^. OGI varies significantly by anatomic site and may be associated with extrusion of intraocular contents^3^. Additionally, the etiologies of OGI vary based on age and demographic characteristics. In pediatric populations, OGI typically results from sharp objects^4^. In contrast, the majority of OGIs occur due to falls (64.4%) and accidental trauma (20%) in patients over 80 years old^5^. With regard to sex, men are more likely to suffer from work-related or construction-related OGI whereas women more frequently experience OGI due to falls^6^. One study found that men tend to have OGI at a younger age (mean age: 44.3), whereas women often experience OGI at older ages (mean age: 65.7)^7^.

Despite advances in surgical care, the prognosis for OGI remains uncertain^8,9^. To predict visual outcomes, Kuhn et al. proposed the ocular trauma score (OTS) to stratify ocular trauma into categories of risk for poor visual prognosis based on characteristics at presentation, which has been shown to be effective in predicting visual outcomes in various single institution studies^10–13^. In brief, presenting visual acuity (VA) and comorbid ocular characteristics are used to generate a classification between 1 and 5, where an OTS Class of 1 has the poorest visual prognosis. However, further assessment is needed to evaluate whether this score generates the highest predictive value on follow-up VA, or if other factors allow for enhanced prognostic capability.

Given the potential variability of OGI characteristics based on demographics and geographic location^14,15^, the purpose of this study was to validate risk factors for poor visual outcomes post-OGI and enhance existing tools to stratify patients’ risk of poor visual prognosis in a large, multi-center sample.

## Methods

This retrospective cohort study used data collected from four participating tertiary ophthalmology centers: Bascom Palmer Eye Institute, Johns Hopkins Wilmer Eye Institute, Wills Eye Hospital, and Massachusetts Eye and Ear. The data was de-identified and combined into a collective database for analysis. This study received Institutional Review Board approval from the University of Miami, Johns Hopkins University, Thomas Jefferson University and Harvard University with a waiver of informed consent due to the retrospective nature of this work. This research adhered to the Tenants of the Declaration of Helsinki.

### Patient selection

Subjects were included in this study based on International Classification of Diseases, Tenth Revision, Clinical Modification codes during clinical visits or Current Procedural Terminology codes for open globe repair between the years 2018 and 2021 (Appendix 1). Data recorded included demographic information, mechanism of injury, date of presentation, presenting visual acuity, clinical exam findings, visual acuity measurement at most recent follow-up and if eyes required enucleation due to the traumatic event. In subjects with OGI in both eyes, the right eye was arbitrarily chosen, while the left eye was discarded for all subsequent analyses. Data related to demographic characteristics, etiology, and ocular exam findings that was not clarified in the patient chart was not recorded and treated as a missing observation in subsequent analyses, while the remainder of the subject’s data was included in the final sample. Regarding visual acuity data, subjects without presenting visual acuity data were included, and the associated OTS were treated as missing observations. Subjects were excluded if no longitudinal visual acuity measurement was recorded at their most recent follow-up visit (except in cases of enucleation).

### Data analysis

A modified OTS, based on criteria established by Kuhn et al.^10^ was calculated for each eye using presenting best corrected distance visual acuity and characteristics of the ocular injury (Table 1). Each eye was assigned raw points (ranging from 0 to 100) corresponding to the best available VA and points were deducted based on associated globe rupture, perforating injury, and relative afferent pupillary defect (RAPD). Unlike the original OTS criteria, points were not deducted for presence of comorbid endophthalmitis or retinal detachment (RD) due to limitations in the collected data. Specifically, clinical notes had ambiguous or incomplete information with regard to these comorbidities, and this information was omitted to reduce bias in the results. Due to this adjustment in scoring, raw scores were then standardized using z-score distributions and assigned an OTS class based on the predefined cutoff values.

**Table 1:** Modified ocular trauma score calculation.

Ocular presentation finding	Raw score adjustment
Presenting visual acuity	
*No light perception*	60
*Hand motion/light perception*	70
*1/200–19/200*	80
*20/200–20/50*	90
*≥ 20/40*	100
Globe rupture	−23
Perforating injury	−14
Rapid afferent pupillary defect	−10
**Total score**	

Subjects’ follow-up VA were transformed to Logarithm of the Minimum Angle of Resolution (LogMAR) values for further analysis. The type of VA was preferentially selected based on the best corrected available visual acuity in the following sequence: (1) with correction, (2) pinhole, or (3) without correction, and used for analysis. Finally, VA at follow-up was classified as a binary variable (better or worse than 20/200; 1.00 logMAR) acting as a cutoff value for monocular blindness, where a VA worse than 20/200 was characterized as a poor outcome.

Relationships between eye-level variables and the presence of monocular blindness at follow-up were assessed using logistic regression models with robust sandwich estimations. Univariable associations were performed to assess the risk of demographic characteristics, etiology and clinical examination findings on monocular blindness at follow-up or if primary enucleation was required. Univariable sub-analyses were also performed on subjects with positive visual outcomes at follow-up (VA better than 20/40). Multivariable analysis examined the risk of OTS categories for monocular blindness, including demographic factors as covariates found to be statistically significant on univariable analysis. All statistical analyses were conducted using Stata software (version 18, StataCorp LP, College Station, TX). The significance level (type 1 error) was established at 0.05 for all statistical evaluations.

### Ethical approval

This study received Institutional Review Board approval from all four participating eye centers.

## Results

A total of 1426 eyes from 1426 subjects were included in this analysis. The baseline characteristics of the patient sample are outlined in Table 2. There was roughly equal representation of subjects from each participating center. The majority of subjects were men (N = 1069, 75.0%) with a mean age of 48.3 years (SD: ± 22.4 years) and more than half self-identified as White (N = 848, 59.5%) for race and non-Hispanic or non-Latino (N = 898, 63.0%) for ethnicity. In addition, 18.0% (N = 246) had no medical insurance at presentation. A majority of eyes (N = 1075, 75.4%) underwent primary closure within the first 24 h of the OGI. The median time from initial presentation to last follow-up was 0.61 years (IQR: 0.17–1.50 years) and was significantly different between patients presenting with and without monocular blindness at extended follow-up (P < 0.001, Wilcoxon Ranksum test).

**Table 2:** Baseline characteristics of the patient sample.

	Follow-up BCVA better than 20/200 (N = 675) N (%)	Follow-up BCVA worse than 20/200 (N = 751) N (%)	Total (N = 1426) N (%)	Test
Age (Mean ± SD)	41.4 ± 19.65	54.5 ± 22.9	48.3 ± 22.4	< 0.001
Sex				< 0.001
Male	543 (80.4)	526 (70.0)	1073 (75.0)	
Female	132 (19.6)	225 (30.0)	357 (25.0)	
Ethnicity				< 0.001
Non-hispanic or Latino	401 (65.3)	497 (78.4)	898 (63.0)	
Hispanic or Latino	213 (34.7)	137 (21.6)	350 (24.5)	
Not reported	–	–	178 (12.5)	
Race				< 0.001
White	418 (77.4)	430 (70.8)	848 (59.5)	
Black	99 (18.3)	169 (27.8)	268 (18.8)	
Asian	15 (2.8)	5 (0.8)	20 (1.7)	
American, Indian or Alaska native	6 (1.1)	1 (0.2)	7 (0.6)	
Hawaiian or Pacific Islander	1 (0.2)	0 (0.0)	1 (0.1)	
More than one race	1 (0.2)	2 (0.3)	3 (0.3)	
Not reported	–	–	279 (19.8)	
Ophthalmology center				0.363
Massachusetts Eye and Ear	183 (27.1)	174 (23.2)	357 (25.0)	
Wills Eye Hospital	171 (25.3)	203 (27.0)	374 (26.2)	
Wilmer Eye Institute	112 (16.6)	137 (18.2)	249 (17.5)	
Bascom Palmer Eye Institute	209 (31.0)	237 (31.6)	446 (31.3)	
Insurance				< 0.001
Uninsured	143 (22.2)	103 (14.4)	246 (18.0)	
Medicaid	65 (10.1)	82 (11.4)	147 (10.8)	
Medicare	83 (12.9)	214 (24.8)	298 (21.8)	
Private	354 (54.9)	319 (44.4)	674 (49.4)	
Not reported			65 (4.6)	
Time to follow-up, years (median [IQR])	0.73 [0.26–1.52]	0.48 [0.11–1.46]	0.6 [0.17–1.50]	< 0.001
Time to surgery				0.329
< 24 h	499 (74.0)	576 (76.7)	1075 (75.4)	
24–48 h	71 (10.5)	58 (7.7)	129 (9.1)	
48–72 h	22 (3.3)	24 (3.2)	46 (3.2)	
> 72 h	83 (12.3)	93 (12.4)	176 (12.3)	

Total sample: N = 1426.

The causes of OGI were identified (Table 3). Most commonly, injuries occurred outside of the home (N = 512, 35.9%) with subjects not wearing eye protection (N = 624, 43.8%). The most frequent injury mechanism was falls (N = 273, 19.3%). Of subjects who reported details on their substance use, the majority did not use drugs or alcohol during the time of injury (N = 614, 43.1%). Sharp objects (N = 641, 45.0%) and metal materials (N = 510, 35.8%) were the most frequently cited cause. Two-hundred and eighty-nine subjects (20.3%) had a history of a prior ophthalmic surgery and 162 (58.1%) of those subjects experienced OGI due to a wound dehiscence from a prior surgical procedure.

**Table 3:** Univariable analysis examining the risk of mechanism of injury on monocular blindness at follow-up.

	Total N (%)	Odds ratio	95% Confidence interval	*P* value
Cause of injury				
Construction	191 (13.4)	1 (base)		
Assault	150 (10.5)	5.78	3.601–9.285	< 0.001
Road traffic accident	55 (3.9)	1.69	0.919–3.118	0.091
Fall	273 (19.3)	6.25	4.150–9.402	< 0.001
Sports-related	46 (3.2)	2.64	1.370–5.093	0.004
Firearm-related	10 (0.7)	8.13	1.675–39.427	0.009
Other	420 (29.4)	1.54	1.075–2.202	0.018
Unspecified	267 (18.7)	–	–	–
Place of injury				
Home	474 (33.2)	1 (base)		
Outside the home	512 (35.9)	0.66	0.516–0.853	0.001
Unspecified	440 (30.9)	–	–	
Object type				
Sharp	641 (45.0)	1 (base)		
Blunt	532 (37.3)	4.50	3.512–5.760	< 0.001
Unspecified	253 (17.7)	–	–	–
Object material				
Vegetative matter	34 (2.4)	1 (base)		
Metal	510 (35.8)	1.51	0.721–3.168	0.274
Fire	21 (1.5)	8.89	2.408–29.783	0.001
Glass	101 (7.1)	1.06	0.463–2.430	0.889
Wood	111 (7.8)	3.00	1.312–6.655	0.009
Stone	83 (5.8)	2.87	1.237–6.646	0.014
Plastic	72 (5.1)	2.93	1.241–6.905	0.014
Other	104 (7.3)	2.22	0.980–5.013	0.056
Combined	2 (0.1)	–	–	–
Unspecified	389 (27.3)	–	–	–
Substance use during injury				
No	614 (43.1)	1(base)		
Yes	49 (3.4)	4.27	2.094–8.717	< 0.001
Unspecified	763 (53.5)	–	–	–
Eye protection during injury				
Yes	67 (4.7)	1 (base)		
No	624 (43.8)	1.59	0.956–2.656	0.074
Unspecified	735 (51.5)	–	–	–
Wound surgical dehisince (Prior ophthalmic surgery: N = 289)				
No	117 (41.9)	1 (base)		
Yes	162 (58.1)	0.888	0.533–1.518	0.652

Total sample: N = 1426.

The ocular examination findings of eyes on initial presentation are detailed in Table 4. Zone I (i.e., cornea and the corneal limbus) injuries were most common (N = 703, 49.3%) with a majority of eyes showing no evidence of associated orbital injury (N = 1115, 78.2%) or eyelid laceration (N = 1194, 83.7%). The rate of traumatic cataract, with or without anterior/posterior capsule violation was 17.5% (N = 249). Commotio retinae was described in 2.2% (N = 32) of eyes and hyphema was noted in 34.2% (N = 488). A RAPD was observed in 19.3% (N = 275) of eyes and iris prolapse in 38.1% (N = 543). Finally, an intraocular foreign body was found in 10.9% (N = 155) of eyes, most commonly in the posterior globe (N = 62, 40.0%).

**Table 4:** Univariable analysis examining the risk of presenting ocular examination features on monocular blindness at follow-up.

	Total N (%)	Odds ratio	95% Confidence interval	*P*-value
Open globe type				
Laceration	781 (54.8)	1 (base)		
Rupture	615 (43.1)	4.09	3.257–5.128	< 0.001
Unspecified	30 (2.1)			
Laceration type (N = 781)				
Penetrating	591 (41.4)	1 (base)		
Perforating	31 (2.2)	1.36	0.657– 2.812	0.408
With intraocular foreign body	155 (10.9)	0.93	0.646–1.135	0.714
Unspecified	649 (45.5)			
IOFB				
None, mean presenting VA = 1.94 [± 1.02] logMAR	1271 (89.1)	1 (base)		
Present, mean presenting VA = 1.23 [± 1.06] logMAR	155 (10.9)	0.47	0.332–0.663	< 0.001
IOFB type (N = 155)				
Non-metal	12 (7.7)	1 (base)		
Metal	123 (79.3)	1.50	0.383–5.870	0.560
Unspecified	20 (12.9)			
IOFB location (N = 155)				
Anterior chamber	40 (25.8)	1 (base)		
Angle	13 (8.4)	0.81	0.265–5.433	0.813
Intralenticular	15 (9.7)	1.00	0.226–3.432	1.000
Vitreous or retina	62 (40.0)	2.76	1.488–9.451	0.005
Anterior and posterior chambers	11 (7.1)	2.29	0.532–9.818	0.266
Unspecified	14 (9.0)			
Injury zone				
Zone 1	703 (49.3)	1 (base)		
Zone 2	378 (26.5)	2.18	1.690–2.812	< 0.001
Zone 3	268 (18.9)	6.20	4.434–8.672	< 0.001
Multiple zones	5 (0.35)	0.39	0.043–3.523	0.403
Unspecified	72 (5.1)			
Eyelid laceration				
None	1194 (83.7)	1 (base)		
Present	179 (12.6)	2.53	1.800–3.568	< 0.001
Unspecified	53 (3.7)			
Relative afferent pupillary defect				
None	731 (51.3)	1 (base)		
Present	275 (19.3)	9.74	6.905–13.739	< 0.001
Unspecified	420 (29.5)			
Hyphema				
None	692 (48.4)	1 (base)		
Filling less than ½ the AC	237 (16.6)	1.63	1.206–2.196	0.001
Filling greater than ½ the AC	251 (17.6)	13.11	8.726–19.705	< 0.001
Unspecified	246 (17.4)			
Lens status				
Normal	359 (25.2)	1 (base)		
Pre-existing cataract	81 (5.7)	2.99	1.808–4.928	< 0.001
Cataract related to trauma with no capsular violation	102 (7.2)	2.18	1.365–3.480	0.001
Traumatic cataract with anterior lens capsule violation	132 (9.3)	1.17	0.739–1.860	0.500
Traumatic cataract with posterior lens capsule violation	15 (1.1)	2.96	1.040–8.399	0.042
Subluxation/dislocation of crystalline lens	39 (2.7)	13.09	5.790–29.596	< 0.001
Pseudophakic with no Subluxation	63 (4.4)	2.70	1.551–4.707	< 0.001
Pseudophakic with subluxation or Dislocation	45 (3.2)	7.48	3.798– 14.733	< 0.001
Other	125 (8.7)	8.35	5.277–13.218	< 0.001
Unspecified	465 (32.6)			
Commotio retinopathy				
None	451 (31.6)	1 (base)		
Involving macula	12 (0.8)	0.70	0.187–2.641	0.602
Involving periphery	18 (1.3)	0.26	0.060–1.164	0.079
Involving macula and periphery	2 (0.1)	–	–	–
Unspecified	943 (66.1)			
Orbit status				
Normal	1115 (78.2)	1 (base)		
Fracture	106 (7.4)	6.74	3.857–11.795	< 0.001
Orbital foreign body	52 (3.7)	1.30	0.742–2.266	0.361
Retrobulbar hemorrhage	17 (1.2)	8.34	1.897–36.653	0.004
Combined	3 (0.2)	–	–	–
Unspecified	133 (9.3)			
Iris prolapse				
None	674 (47.3)	1 (base)		
Present	543 (38.1)	2.16	1.718–2.724	< 0.001
Unspecified	209 (14.7)			
VA at Presentation (Mean ± SD)	1.86 ± 1.04	5.54	4.423–6.942	< 0.001

Total sample: N = 1426.

*VA* Visual acuity; *AC* Anterior chamber.

Univariable analysis of the demographic characteristics in this cohort revealed several associations with worse visual prognosis, presented in Table 5. Older age at presentation (OR: 1.32 per decade older [1.26–1.40]; *P* < 0.001) and women (OR: 1.76 [1.38–2.25]; *P* < 0.001) both increased risk of blindness at follow-up. The risk of enucleation (*P* = 0.338) did not significantly differ between pediatric and adult subjects, whereas the overall risk of blindness did (*P* = 0.007). Black subjects were 66% (OR: 1.66 [1.25–2.20]; *P* < 0.001) more likely to present with blindness at follow-up compared with White subjects. Subjects of Hispanic ethnicity had a lower risk of blindness (OR: 0.52; 0.40–0.67; *P* < 0.001) than non-Hispanics. A greater time to follow-up was associated with a decreased risk of blindness (OR: 0.91 [0.82–1.00], *P* = 0.047).

**Table 5:** Univariable analysis examining the risk of patient baseline characteristics on monocular blindness at follow-up.

	Total N (%)	Odds ratio	95% Confidence interval	*P* value
Age (Mean ± SD)	48.28 ± 22.39	1.32	1.258–1.395	< 0.001
Sex				
Male	1073 (75.0)	1 (base)		
Female	357 (25.0)	1.76	1.376–2.251	< 0.001
Ethnicity				
Non-hispanic or Latino	898 (63.0)	1 (base)		
Hispanic or Latino	350 (24.5)	0.52	0.403–0.668	< 0.001
Not reported	178 (12.5)	–	–	–
Race				
White	848 (59.5)	1 (base)		
Black	268 (18.8)	1.66	1.251–2.201	< 0.001
Asian	20 (1.7)	0.32	0.116–0.900	0.031
American, Indian or Alaska Native	7 (0.6)	0.16	0.019–1.353	0.093
Hawaiian or Pacific Islander	1 (0.1)	–	–	–
More than 1 race	3 (0.3)	1.94	0.175–21.545	0.588
Not reported	279 (19.8)			
Insurance				
Private	246 (18.0)	1 (base)		
Medicaid	147 (10.8)	1.40	0.978–2.004	0.066
Medicare	298 (21.8)	2.86	2.130–3.884	< 0.001
Uninsured	674 (49.4)	0.80	0.595–1.074	0.137
Missing	65 (4.6)	–	–	–
Time to follow up, years (Mean ± SD)	0.6 [0.17–1.50]	0.91	0.821–0.999	0.047
Time to surgery				
< 24 h	1075 (75.4)	1 (base)		
24–48 h	129 (9.1)	0.71	0. 490–1.022	0.065
48–72 h	46 (3.2)	0.95	0.523–1.706	0.851
> 72 h	176 (12.3)	0.97	0.705–1.335	0.855

Total sample: N = 1426.

Different mechanisms of injury were found to predict the likelihood of blindness assessed on univariable analysis in Table 3. Firearm-related OGI was more likely to be associated with blindness (OR: 8.13 [1.68–39.43]; *P* = 0.009). Assault (OR: 5.78 [3.60–9.29]; *P* < 0.001), falls (OR: 6.25 [4.15–9.40]; *P* < 0.001) and sports-related trauma (OR: 2.64 [1.37–5.09]; *P* = 0.004) also had increased risk of blindness when compared to construction-related injury. Blunt trauma was over 4 times more likely to yield poor visual prognosis than sharp trauma (OR: 4.50 [3.51–5.76]; *P* < 0.001). Injuries outside the home had a decreased risk (OR: 0.66 [0.52–0.85]; *P* < 0.001) of blindness relative to trauma occurring inside the home. Fire or blast-related materials carried the highest risk of blindness (OR: 8.89 [2.41–29.78]; *P* = 0.001). Subjects who reported use of substances during the injury (OR: 4.27 [2.09–8.71]; *P* < 0.001) had a significantly increased risk of blindness.

We also evaluated ocular examination findings on presentation and their associated risk of blindness at follow-up using univariable analysis (Table 4). Injuries to Zone III (i.e., an anatomic area that extends 5 mm and beyond posterior to the limbus) carried the highest risk of blindness (OR: 6.20 [4.43–8.67]; *P* < 0.001) compared with Zone I injury. Eyes with subluxation or dislocation of the lens (OR: 13.09 [5.79–29.60]; *P* < 0.001), hyphema filling more than half the anterior chamber (OR: 13.11 [8.73–19.71] *P* < 0.001), RAPD (OR: 9.74 [6.91–13.74]; *P* < 0.001), retrobulbar hemorrhage (OR: 8.34 [1.90–36.65]; *P* = 0.004), eyelid lacerations (2.53 [1.80–3.57]; *P* < 0.001), and iris prolapse (OR: 2.16 [1.72–2.72]; *P* < 0.001) had increased risk of poor visual outcome at follow-up compared to eyes without these exam findings, respectively. Subjects with intraocular foreign body (IOFB) had decreased odds of blindness (OR: 0.47 [0.33–0.66]; *P* < 0.001); but among subjects with IOFB, localization to the posterior segment carried approximately 3.8 times higher odds of blindness (OR: 3.75 [1.49–9.45]; *P* = 0.005) than those localized to the anterior chamber. However, subjects without IOFB had significantly worse presenting VA (mean = 1.94 [± 1.02] logMAR) than subjects with IOFB (mean = 1.23 [± 1.06] logMAR) at presentation (*P* < 0.001).

Based on univariable analysis, the modified OTS classification was found to be the strongest predictor of a poor visual outcome at follow-up. Eyes with OTS Class 1 had over 38 times higher risk of blindness (OR: 38.35 [21.33–68.93]; *P* < 0.001), using OTS Class 5 as the reference. With regard to enucleation, OTS Class 1 had the greatest associated risk on univariable analysis (OR: 8.28 [2.54–26.93]; *P* < 0.001). No cases of enucleation were identified in subjects with OTS Class 4 or 5. Finally, multivariable analysis demonstrated that low OTS, including age, sex, race, and time to follow-up visit as covariates, moderately predicted poor visual outcomes (R^2^ = 0.275, *P* < 0.001) (Table 6). OTS Class 1 (OR: 23.88 [16.44–45.85]; *P* < 0.001) remained significantly associated with higher odds of blindness at extended follow-up. In addition, increased age (OR: 1.17 per decade older [1.08–1.26]; *P* < 0.001), longer time to follow-up (OR: 0.85 per year [0.73–0.98]; *P* = 0.029), and Black race (OR: 1.47 [1.01–2.14]; *P* = 0.045) were associated with the odds of blindness.

**Table 6:** Multivariable analysis of the risk of OTS adjusted for demographic characteristics and time to follow-up as a prediction of monocular blindness at follow-up.

	Odds ratio	95% Confidence interval	*P* value
OTS category			
OTS class 1	23.88	12.436–45.851	< 0.001
OTS class 2	19.30	9.845–37.825	< 0.001
OTS class 3	3.85	1.997–7.408	< 0.001
OTS class 4	1.12	0.474–2.622	0.802
OTS class 5	1 (base)		
Time to follow up (years)	0.85	0.730–0.983	0.029
Age (decades)	1.17	1.081–1.259	< 0.001
Sex			
Male	1 (base)		
Female	1.02	0.696–1.491	0.923
Race			
White	1 (base)		
Black	1.47	1.009–2.144	0.045
Asian	0.33	0.107–1.014	0.053
American, Indian or Alaska Native	0.21	0.027–1.619	0.134
Hawaiian or Pacific Islander	–	–	–
More than 1 race	–	–	–

Total sample: N = 986; R^2^ = 0.275.

*OTS* Ocular trauma score.

## Discussion

In this study, we examined the relationship between patient demographic characteristics, etiology of ocular trauma, and associated clinical examination findings with long-term visual outcomes. This was a multi-centered study, and to the authors’ knowledge, represents one of the largest cohorts reported with granular patient-level data. We identified various factors associated with higher odds of poor visual prognosis across this generalizable sample that may inform future risk assessment tools.

The visual outcomes of OGI can be influenced by demographic risk factors^7^. Our results align with reports that highlight racial disparities with poor visual outcomes following OGI in Black patients^16^. In our analysis, patients who self-identified as Black were at 66% greater odds of blindness as compared to White subjects. Despite relatively poorer outcomes, when compared to the overall cohort, Black patients presented with similar OTS, had comparable lengths of time from injury to surgical intervention, and had similar lengths of time to follow-up visits. Of note, subjects of Black race were found to have different rates of health insurance status compared to the remainder of the cohort. These findings warrant further exploration, as disparities in treatment outcomes for Black patients have been observed in other ophthalmology subspecialties^17^.

There have been mixed findings regarding the relationship between how the time interval between OGI and primary closure relates to visual outcomes^11,18,19^. In our study, 75.4% of cases received surgical intervention within 24 h of the traumatic event, with no increased risk of poor visual outcome with operations completed after 24 h. Similarly, when assessing the likelihood of a positive visual result (< 20/40), subjects who received surgery within 24 h did not have greater probability of better outcomes based on univariable analysis. Other studies have found that improved visual outcomes with cases operated within 12 h of injury while delayed surgical repair after 24 h is thought to increase the risk of postoperative endophthalmitis and other sequelae of delayed treatment^19^. Across our study population, the majority of patients were operated on within 24 h, which may have limited evaluation of the effect of delayed primary closure on visual outcomes.

The mechanism of injury for OGI can vary significantly depending on the population studied and have different associated risks for visual prognosis^11,20^. A strength of this study is that it included the majority of dedicated ocular emergency departments existing in the United States, and therefore may act as a general sample to examine the etiology of OGI. The poorest visual outcomes and risk of enucleation occurred after injury due to firearms, likely due to multiple associated injuries to the ocular, orbital, or visual system pathways, that have been characterized^15^. In agreement with previous studies, ocular trauma leading to globe rupture was found to be a significant predictive factor for the risk of blindness^13,21^.

In our analysis, a worse modified OTS at presentation was strongly associated with increased odds of blindness at follow-up and/or enucleation. The OTS is a validated tool that has demonstrated a highly predictive capability of visual outcomes following repair in OGI^22,23^. The main variables used in the OTS are VA on presentation and associated ocular injuries including globe rupture, endophthalmitis, perforating injury, RD, and RAPD^10^. While RD and endophthalmitis were not used in this study, it should be noted that endophthalmitis and RD individually are strong predictors of visual outcomes^12^. However, even without these factors, a worse modified OTS classification remained significantly associated with higher odds of blindness, demonstrating that other included OTS criteria at presentation (i.e., perforating injury, RAPD, and OGI) also play an important role in visual prognosis. In terms of examination findings, more severe ocular traumas leading to RAPD, retrobulbar hemorrhage, large hyphema, prolapsed iris, lens subluxation, and Zone III injury were factors associated with worse visual outcomes and have been identified in other studies^12,13^.

Interestingly, while previous reports found that IOFB had no impact on visual outcome^12,24^, we observed that eyes with the presence of IOFB had better visual outcomes in this study. This finding should be evaluated with caution, since subjects with IOFB in our sample presented with significantly less severe VA loss compared with eyes without IOFB. With VA at presentation being one of the most important predictive factors for low follow-up visual outcome in our analysis and others^25^, it is possible that this difference influenced the results. However, among subjects with an IOFB, a posterior location in the vitreous or retina had the highest risk of blindness compared with anterior segment IOFB and further supports existing literature^26^.

Forty-eight eyes underwent primary enucleation in our cohort, representing 3.4% of the total sample. This rate of primary enucleation falls within the previously reported values between 0.0 and 7.4%^27–30^. The most common cause of injury in this subgroup of eyes in our analysis was assault, which has been associated with increased risk of globe removal^31^. The clinical risk factors for enucleation following OGI have been assessed^30^, and can include wound length greater than 10 mm, uveal prolapse, higher Zone of injury, IOFB, and RAPD. Of the variables collected in our analysis, higher Zone of injury, RAPD, and iris prolapse were also found to be associated with greater risk of enucleation, while no relationship was observed with IOFB.

This study has several limitations. Although great effort was made to include all subjects seen in each site for OGI during the study period, this retrospective analysis of visual outcomes post-OGI excluded patients lost to follow-up or without longitudinal visual acuity data. In addition, subjects were treated at the discretion of the attending physician, and there was no control for interventions that subjects received between OGI and follow-up visit, limiting our analysis of treatment-related risk factors. This effect may in part be observed in the relationship identified between the increased time to follow-up visit and the decreased likelihood of blindness, as patients followed for increased time periods may receive additional medical and surgical interventions. In addition, patients who had poor visual outcomes following their ocular trauma were more likely to not continue extended follow-up visits. The data in this study relies on the use of electronic medical records and includes patient-reported details, which may be subject to bias. In addition, while this cohort represents four tertiary ophthalmology centers in different locations in the United States, these results may not be generalizable to a broader population. Finally, the OTS presented in this analysis is not based on original scoring criteria, and omit the presence of endophthalmitis and RD.

In conclusion, we found that patient baseline characteristics, such as older age, women, and Black race, a modified OTS, and ocular exam findings were predictive of worse visual outcomes. This sample represents one of the largest generalizable cohorts examined for patients treated in dedicated ocular emergency rooms in the United States. Although outcomes in OGI have improved with better access to care and advancements in surgical techniques, our findings reveal additional pertinent risk factors for patients with OGI that may be used to stratify risk for poor visual outcome. Future prospective studies should examine additional risk factors for patients presenting with ocular trauma and potential causes of racial disparity observed in this analysis.

### Supplementary Information

Supplementary Information.

## Data Availability

Data can be made available upon request. Requests can be made to Jason Greenfield at:—jason.greenfield@med.miami.edu.
