# Supplementary material for: A multi-center analysis of visual outcomes following open globe injury
Source: Sci Rep. 2024 Jul 18;14:16638. doi: 10.1038/s41598-024-67564-y (PMC11258120; doi:10.1038/s41598-024-67564-y)
Supplement: Supplementary file 1 — Supplementary Information. [file 41598_2024_67564_MOESM1_ESM.docx]

**Extracted CPT and ICD10 Codes**

| **Condition** | **CPT** |  |
| --- | --- | --- |
| Open globe, disruption of operative incision | 65275 | Repair of laceration; cornea, nonperforating, with or without removal foreign body |
| Open globe, disruption of operative incision | 65280 | Repair of laceration; cornea and/or sclera, perforating, not involving uveal tissue |
| Open globe, disruption of operative incision | 65285 | Repair of laceration; cornea and/or sclera, perforating, with reposition or resection of uveal tissue |
| Open globe, disruption of operative incision | 65286 | Repair of laceration; application of tissue glue, wounds of cornea and/or sclera |
| Open globe, disruption of operative incision | 66250 | Revision or repair of operative wound of anterior segment, any type, early or late, major or minor procedure |
| Intraocular foreign body | 65235 | Removal of foreign body, intraocular; from anterior chamber of eye or lens |
| Intraocular foreign body | 65260 | Removal of foreign body, intraocular; from posterior segment, magnetic extraction, anterior or posterior route |
| Intraocular foreign body | 65265 | Removal of foreign body, intraocular; from posterior segment, nonmagnetic extraction |
| Intraocular foreign body | 67413 | Orbitotomy without bone flap (frontal or transconjunctival approach); with removal of foreign body |
|  |  |  |
|  |  |  |
| **Diagnosis** | **ICD10** |  |
| Ocular laceration and rupture with prolapse or loss of intraocular tissue | S05.2 |  |
| Ocular laceration and rupture with prolapse or loss of intraocular tissue, unspecified eye | S05.20 |  |
| …… initial encounter | S05.20XA |  |
| …… subsequent encounter | S05.20XD |  |
| …… sequela | S05.20XS |  |
| Ocular laceration and rupture with prolapse or loss of intraocular tissue, right eye | S05.21 |  |
| …… initial encounter | S05.21XA |  |
| …… subsequent encounter | S05.21XD |  |
| …… sequela | S05.21XS |  |
| Ocular laceration and rupture with prolapse or loss of intraocular tissue, left eye | S05.22 |  |
| …… initial encounter | S05.22XA |  |
| …… subsequent encounter | S05.22XD |  |
| …… sequela | S05.22XS |  |
| Ocular laceration without prolapse or loss of intraocular tissue | S05.3 |  |
| Ocular laceration without prolapse or loss of intraocular tissue, unspecified eye | S05.30 |  |
| …… initial encounter | S05.30XA |  |
| …… subsequent encounter | S05.30XD |  |
| …… sequela | S05.30XS |  |
| Ocular laceration without prolapse or loss of intraocular tissue, right eye | S05.31 |  |
| …… initial encounter | S05.31XA |  |
| …… subsequent encounter | S05.31XD |  |
| …… sequela | S05.31XS |  |
| Ocular laceration without prolapse or loss of intraocular tissue, left eye | S05.32 |  |
| …… initial encounter | S05.32XA |  |
| …… subsequent encounter | S05.32XD |  |
| …… sequela | S05.32XS |  |
|  |  |  |
| Penetrating wound of orbit with or without foreign body | S05.4 |  |
| Penetrating wound of orbit with or without foreign body, unspecified eye | S05.40 |  |
| …… initial encounter | S05.40XA |  |
| …… subsequent encounter | S05.40XD |  |
| …… sequela | S05.40XS |  |
| Penetrating wound of orbit with or without foreign body, right eye | S05.41 |  |
| …… initial encounter | S05.41XA |  |
| …… subsequent encounter | S05.41XD |  |
| …… sequela | S05.41XS |  |
| Penetrating wound of orbit with or without foreign body, left eye | S05.42 |  |
| …… initial encounter | S05.42XA |  |
| …… subsequent encounter | S05.42XD |  |
| …… sequela | S05.42XS |  |
|  |  |  |
| Penetrating wound with foreign body of eyeball | S05.5 |  |
| Penetrating wound with foreign body of unspecified eyeball | S05.50 |  |
| …… initial encounter | S05.50XA |  |
| …… subsequent encounter | S05.50XD |  |
| …… sequela | S05.50XS |  |
| Penetrating wound with foreign body of right eyeball | S05.51 |  |
| …… initial encounter | S05.51XA |  |
| …… subsequent encounter | S05.51XD |  |
| …… sequela | S05.51XS |  |
| Penetrating wound with foreign body of left eyeball | S05.52 |  |
| …… initial encounter | S05.52XA |  |
| …… subsequent encounter | S05.52XD |  |
| …… sequela | S05.52XS |  |
|  |  |  |
| Penetrating wound without foreign body of eyeball | S05.6 |  |
| Penetrating wound without foreign body of unspecified eyeball | S05.60 |  |
| …… initial encounter | S05.60XA |  |
| …… subsequent encounter | S05.60XD |  |
| …… sequela | S05.60XS |  |
| Penetrating wound without foreign body of right eyeball | S05.61 |  |
| …… initial encounter | S05.61XA |  |
| …… subsequent encounter | S05.61XD |  |
| …… sequela | S05.61XS |  |
| Penetrating wound without foreign body of left eyeball | S05.62 |  |
| …… initial encounter | S05.62XA |  |
| …… subsequent encounter | S05.62XD |  |
| …… sequela | S05.62XS |  |
